# Shear Stress Modulation of IL-1β-Induced E-Selectin Expression in Human Endothelial Cells

**DOI:** 10.1371/journal.pone.0031874

**Published:** 2012-02-24

**Authors:** Ryan B. Huang, Omolola Eniola-Adefeso

**Affiliations:** Department of Chemical Engineering, University of Michigan, Ann Arbor, Michigan, United States of America; Michigan State University, United States of America

## Abstract

**Background:**

Endothelial cells (ECs) are continuously exposed to hemodynamic forces imparted by blood flow. While it is known that endothelial behavior can be influenced by cytokine activation or fluid shear, the combined effects of these two independent agonists have yet to be fully elucidated.

**Methodology:**

We investigated EC response to long-term inflammatory cues under physiologically relevant shear conditions via E-selectin expression where monolayers of human umbilical vein ECs were simultaneously exposed to laminar fluid shear and interleukin-1ß (shear-cytokine activation) in a parallel plate flow chamber.

**Results and Conclusion:**

Naïve ECs exposed to shear-cytokine activation display significantly higher E-selectin expression for up to 24 hr relative to ECs activated in static (static-cytokine). Peak E-selectin expression occurred after 8–12 hr of continuous shear-cytokine activation contrary to the commonly observed 4–6 hr peak expression in ECs exposed to static-cytokine activation. Cells with some history of high shear conditioning exhibited either high or muted E-selectin expression depending on the durations of the shear pre-conditioning and the ensuing shear-cytokine activation. Overall, the presented data suggest that a high laminar shear enhances acute EC response to interleukin-1ß in naïve or shear-conditioned ECs as may be found in the pathological setting of ischemia/reperfusion injury while conferring rapid E-selectin downregulation to protect against chronic inflammation.

## Introduction

Located at the interface between the vascular wall and the bloodstream, the endothelium (a monolayer of endothelial cells) plays a critical role in several physiological processes including angiogenesis, thrombosis, regulation of blood pressure, and inflammation. Endothelial cell (EC) response has also been implicated in the pathogenesis and pathology of many human diseases. In particular, the EC chronic inflammation response is known to have a prominent role in cancer metastasis and atherogenesis (development of plaque in arteries), a precursor to coronary artery disease [1], [2], [3], [4], [5], [6]. The differential response of ECs to fluid shear stress and various chemical agonists such as tumor necrosis factor-α (TNF-α) and interleukin-1β (IL-1β) results in the differential surface expression of various leukocyte adhesion molecules (LAMs) by the endothelium (*e.g.* selectins, ICAM-1 and VCAM-1) and is critical to the balance between healthy and pathogenic inflammation response [7], [8], [9], [10]. The ability to discriminate between healthy and diseased tissue through EC adhesion molecule expression patterns will have profound consequences for developing diagnostic tools and targeted therapeutic for the treatment of several human diseases. Despite this potential, endothelial behavior under chronic inflammation has yet to be fully elucidated, even after decades of research. While more complex *in vitro* assays to model EC inflammation response have been developed, many of these fail to encompass the true physiological conditions under which inflammation occurs.

To date, most published works on inflammation research have focused on *in vitro* endothelial response to various inflammatory agonist (*e.g.* TNF-α) and antagonist (*e.g.* statins) in static cultures [11], [12], [13], [14], [15] or to mechanical stresses imparted by shear flow of various type (steady or disturbed) and magnitude [16], [17], [18], [19], [20]. Limited works have been done to understand the combined effect of both chemical and mechanical stimuli – a more appropriate representation of the *in vivo* occurrence of inflammatory response. Works that have explored these models have mostly done so in a less than physiological manner, *e.g.* ECs are first exposed to fluid shear stress (*i.e.* pre-conditioned) and then subjected to chemical stimuli under static conditions [21]. Additionally, simultaneous shear-cytokine induced EC response is typically observed in limited time frames [22], [23], [24]. TNF-α has also been the major focus of existing EC shear-cytokine activation studies though other cytokines are known to be a key regulator of inflammation response, *e.g.* IL-1β has been implicated in the pathology of several human diseases, including chronic autoimmune diseases [25], Alzheimer's disease [26], and metabolic syndromes such as atherosclerosis, chronic heart failure, and diabetes [27], [28]. Finally, the majority of these studies have also placed emphasis on understanding ICAM-1 expression rather than E-selectin perhaps due to previous reports of the latter's insensitivity to shear stress [22], [29]. The interaction of EC surface-expressed selectins (E and P-selectin) with their counter receptors (sLe^X^ and PSGL-1, respectively) expressed on leukocyte surfaces, however, is critical to the initiation of leukocyte margination – a hallmark of inflammation response [30]. Moreover, E-selectin is often associated with leukocyte recruitment and disease progression in many chronic inflammatory diseases, *e.g.* increased levels of E-selectin and high leukocyte infiltration is found in rheumatoid arthritis, chronic lung infections and several cardiovascular diseases [31], [32], [33], [34].

In general, understanding longer-term endothelial response to inflammatory cues via surface expression of LAMs would provide invaluable insight into its role in pathological conditions and offer new opportunities for treatment. Thus, the present study aimed to explore the temporal cell-surface expression of E-selectin by human umbilical vein endothelial cells (HUVEC) in response to IL-1β stimulation under laminar shear conditions. We specifically focused on cell-surface (apical) E-selectin expression over time since this directly translates to the temporal level of leukocyte margination [35], [36]. We found that E-selectin is expressed on HUVEC surface in previously unreported patterns where the magnitude and pattern of expression is simultaneously dependent on the magnitude of the imposed fluid shear, the shear history of the ECs, and the length of exposure to chemical stimuli.

## Materials and Methods

### Ethics Statement

All human protocols were approved by the University of Michigan (UM) Internal Review Board (IRB protocol numbers HUM00013973 and HUM00026898) and in line with the standards set by the Helsinki Declaration. A written informed consent approved by the UM IRB was obtained from all participants involved in the study prior to blood collection. Data collection and analyses were performed anonymously.

### Cell Culture

All cell culture reagents were obtained from Invitrogen (Carlsbad, CA) unless otherwise noted. HUVECs were isolated from fresh umbilical cords following a modified Jaffe protocol [37], [38]. Cords were collected from Mott's Children Hospital (Ann Arbor, MI) following an Internal Review Board (IRB) exempt protocol, and HUVECs used in all assays were pooled from at least 3 donors. Isolated cells were collected and plated in 0.2% (w/v) gelatin (Sigma Aldrich, St. Louis, MO) surface-treated tissue culture flasks and cultured until confluent in HUVEC media that consisted of Medium 199 supplemented with 10% (v/v) fetal bovine serum (HyClone, Logan, Utah), 10% (v/v) bovine calf serum (HyClone, Logan, Utah), 1% 250 µg/mL fungizone, 1% 5,000 U/mL penicillin/5,000 µg/mL streptomycin, 1% 200-mM L-glutamine, 1% 10 mg/mL heparin, 1% 1 M HEPES buffer and 50 µg/ml endothelial cell growth supplement (BD Biosciences, Franklin Lakes, NJ).

For static and shear flow experiments, HUVECs were trypsinized (0.25% trypsin-EDTA) and subcultured onto 30 mm glass coverslips (Warner Instruments, Hamden, CT) coated with 1% w/v gelatin cross-linked with glutaraldehyde (Polysciences, Warrington, PA) as previously described [39]. Coverslips were incubated for at least 36 hr in a humidified, 5% CO_2_, 37°C environment prior to use. HUVEC monolayers cultured as described can maintain their integrity when exposed to laminar flow of up to 20 dynes cm^−2^ [38]. HUVECs were not used in experiments beyond passage 2.

### Parallel Plate Flow Chamber Setup

A straight channel parallel plate flow chamber (PPFC) (GlycoTech, Gaithersburg, MD) was used for all shear studies [40]. Briefly, a silicon rubber gasket with a rectangular cutout was attached to an acrylic flow chamber deck with inlet and outlet flow ports. A glass coverslip with confluent HUVEC monolayer was placed over the gasket and vacuum-sealed such that the coverslip formed the bottom plate of the flow chamber. A rectangular cutout in the gasket defined the flow channel with a height of 254 µm. The PPFC was connected via 1.8 mm inner diameter Tygon tubing to inlet and outlet media reservoirs. A height difference between reservoirs determined the flow rate through the PPFC; a programmable peristaltic pump (Ismatec, Germany) was used to recirculate media to the inlet reservoir. The entire apparatus was maintained in a humidified 5% CO_2_, 37°C chamber built around a Nikon TE 2000-S inverted microscope.

### Activation of Endothelial Cells

For static conditions, HUVEC monolayers were exposed to a single dose of 0.1 ng ml^−1^ of recombinant human interleukin-1ß (IL-1ß – Fitzgerald Industries, Concord, MA) for the desired period of time (up to 24 hr). Preliminary experiments showed maximal E-selectin expression with an IL-1ß concentration of 0.01 ng ml^−1^ at the 4 hr activation mark under static conditions.

For shear conditions, HUVEC monolayers were exposed to a fixed wall shear (in the range of 1–20 dyn cm^−2^) via flow of cell culture media in either the absence or presence of IL-1ß for a given period of time up to 24 hr. The wall shear stress (dyn cm^−2^) in the flow chamber was computed as previously described [38].

### Quantification of HUVEC E-selectin Surface Expression

Coverslips with HUVEC monolayers from static and flow assays were washed with DPBS^++^ and fixed via exposure to 4% w/v paraformaldehyde for 10 min at 4°C. Cells were washed twice with DPBS^++^ and incubated with 0.2 mg ml^−1^ fluorescein-conjugated monoclonal anti-human E-selectin antibody (BBIG-E5 clone, R&D Systems, Minneapolis, MN) at 4°C for at least 1 hr to label surface-expressed protein only – no cell permeabilizing agent was used. After labeling, monolayers were affixed to flow chambers in their original orientation in order to maintain a consistent imaging depth; chambers were flushed with DPBS^++^ to clear cell surface debris and unbound antibodies. Monolayer surface E-selectin expression was imaged on a fluorescent microscope (Nikon ET 2000 S) at 485/527 nm (ex/em) and 2000 ms exposure using a Photometrics CoolSNAP EZ (Photometrics, Tucson, AZ) mounted on a Sony CCD sensor digital camera; images were captured using MetaMorph software (Downingtown, PA). A representative fluorescent image of EC E-selectin expression is shown in Figure S1. Relative fluorescent intensities (RFI) were collected for five images per monolayer near the PPFC flow centerline at least 1 cm from the flow inlet. To convert RFI values to antibody concentration, average fluorescent intensities for 0.2, 0.1, 0.05, and 0.00 µg ml^−1^ solutions of the anti-human E-selectin antibody were measured on a calibration monolayer for a linear regression fit. Measured RFI values for each monolayer were then converted to E-selectin sites per µm^2^ (assuming 1∶1 binding ratio of antibody to receptor).

### Neutrophil Binding Assays

Human blood was collected from donors via venipuncture into a syringe containing citrate anticoagulant (acetate-citrate-dextrose). Red blood cells (RBCs) were separated from whole blood by sedimentation using a 6% (w/v) dextran solution. Neutrophils were isolated via the standard Ficoll gradient as previously described [39]. Isolated neutrophils were washed with DPBS^−^ at room temperature and used within 2 hr of isolation. Neutrophil adhesion to IL-1ß treated (up to 24 hr) HUVEC monolayers under static- or shear-cytokine conditions were assessed in the PPFC described above. An activated monolayer was loaded onto the flow deck, and freshly isolated human neutrophils in DPBS^++^ with 1% (w/v) human serum albumin (1×10^6^ cells ml^−1^) were perfused over the monolayer at 2 dyn cm^−2^. Binding density was calculated as the number of adherent or rolling neutrophils per mm^2^ after 2 minutes of shear.

### Statistical Methods

All experiments were performed in at least triplicate; averages were calculated and standard errors are shown on data figures unless otherwise noted. Significance was determined using student's t-test for one-on-one comparisons and one-way ANOVA with a 95% confidence interval and Bonferroni post-test using GraphPad Prism Software (San Diego, CA) for comparison between groups.

## Results

### HUVEC Response to Simultaneous Exposure to IL-1ß and Laminar Shear

**Figure 1:**
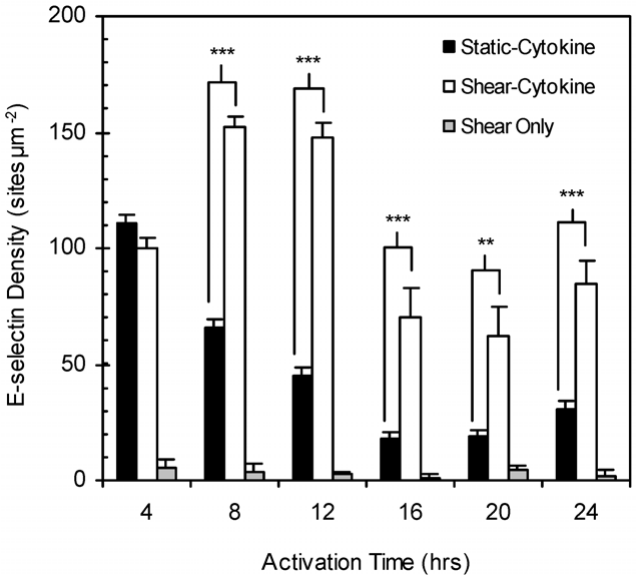
E-selectin expression (sites µm^−2^) in static-cytokine, shear-cytokine and shear only activated monolayers. HUVEC monolayers were exposed to laminar shear alone at 10 dyn cm^−2^ (gray) or activated with 0.1 ng ml^−1^ IL-1ß either under static culture (solid) or simultaneously exposed to fluid shear stress (clear) at 10 dyn cm^−2^. “NS” indicates no significance, “**” *p*<0.01, and “***” *p*<0.001.

Figure 1 shows the E-selectin surface expression density for HUVEC monolayers activated with shear alone (10 dyn cm^−2^), IL-1ß in static (static-cytokine hereafter), or IL-1ß in shear flow (shear-cytokine; 10 dyn cm^−2^). Naïve monolayers exposed to a single dose of static-cytokine activation exhibited increased E-selectin expression from basal expression (untreated cells; ∼9.0 sites µm^−2^) to levels as high as 111 sites µm^−2^ at 4 hr (peak expression). E-selectin expression subsequently decreased to values only slightly significant from baseline by 16 hr (*p* = 0.030; unpaired t-test). At 24 hr of static-cytokine activation, expression slightly rebounded to a level 3.4 times higher than baseline expression (*p* = 0.0057; unpaired t-test). When IL-1ß activation was done in the simultaneous presence of laminar shear at 10 dyn cm^−2^ (shear-cytokine), HUVEC monolayers expressed significantly higher levels of E-selectin than that observed for static-cytokine activation at all time points from 8 to 24 hr. The same level of expression was recorded at 4 hr for both shear-cytokine and static-cytokine treated monolayers. E-selectin density on shear-cytokine activated cells was also not significantly different at 4 and 24 hr of activation time whereas the 24 hr level for static-cytokine activated cells was 3.5 fold lower than their corresponding 4 hr expression. As shown in Figure 1, monolayers exposed to laminar shear alone displayed minimal E-selectin expression not significant from the basal expression for up to 24 hr.

**Figure 2:**
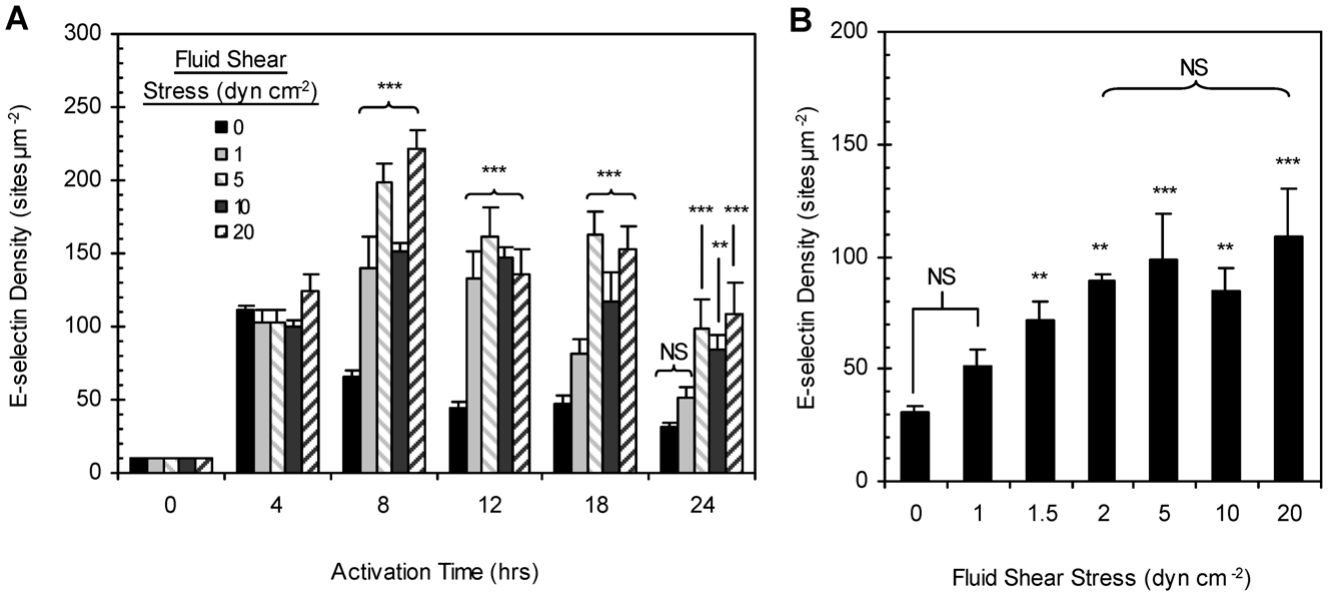
E-selectin expression (sites µm^−2^) on 0–20 dyn cm^−2^ shear-cytokine activated monolayers. (A) Contour plot of E-selectin expression by HUVEC monolayers activated with 0.1 ng ml^−1^ IL-1ß either under static conditions (no fluid shear) or simultaneously exposed to 1, 5, 10 or 20 dyn cm^−2^ of laminar fluid shear at activation periods ranging from 0 to 24 hr. *Note*: This is a plot of data collected at discrete time points and shear levels as indicated on the *x* and *y* axes, respectively. (B) Expression at 24 hr only. “NS” indicates no significance, “*” *p*<0.05, “**” *p*<0.01, and “***” *p*<0.001 when compared to static-cytokine activation at the same time point.

An extension of the fluid shear stress studied to 5 and 20 dyn cm^−2^ showed that the peak E-selectin expression for all shear-cytokine activation shifted to the 8 to 12 hr range with the same magnitude of expression over static-cytokine activation as shown in Figure 2A. Surprisingly, even at the low shear magnitude of 1 dyn cm^−2^, peak expression also shifted to 8–12 hr with levels comparable to that observed at 10 dyn cm^−2^. Specifically, the E-selectin expression by monolayer exposed to 1 dyn cm^−2^ of shear-cytokine activation was not significant at the 8 hr mark from values measured for monolayers exposed to 5 and 10 dyn cm^−2^ at the same time point (one-way Anova). Expression levels also remained elevated relative to static-cytokine for most activation times greater than 4 hr at this low shear-cytokine activation. Overall, the 24 hr expression in shear-cytokine activated monolayers exposed to at least 5 dyn cm^−2^ of shear remained 2–3 folds higher than the 24 hr expression measured for static-cytokine treated ECs and were comparable to the expression level at 4 hr of static-cytokine activation. The E-selectin density at 24 hr for 1 dyn cm^−2^ of shear-cytokine activation was not significant from the expression at 24 hr for static-cytokine. Thus, we examined additional shear stress magnitudes between 1 and 5 dyn cm^−2^ for the 24 hr activation time point. As shown in Figure 2B, a linear correlation between E-selectin density and shear magnitude was observed from static-cytokine activation up to 2 dyn cm^−2^ of shear-cytokine activation. The selectin densities measured for shear magnitudes from 2 to 20 dyn cm^−2^ shear-cytokine activation were not significant from each other. Overall, endothelial monolayer integrity was maintained for up to 24 hr in the range of laminar shear stresses studied here (see Figure S2), and all monolayer exposed to shear (alone or with cytokine) were found to elongate in the direction of flow as previously reported in the literature [41], [42].

**Figure 3:**
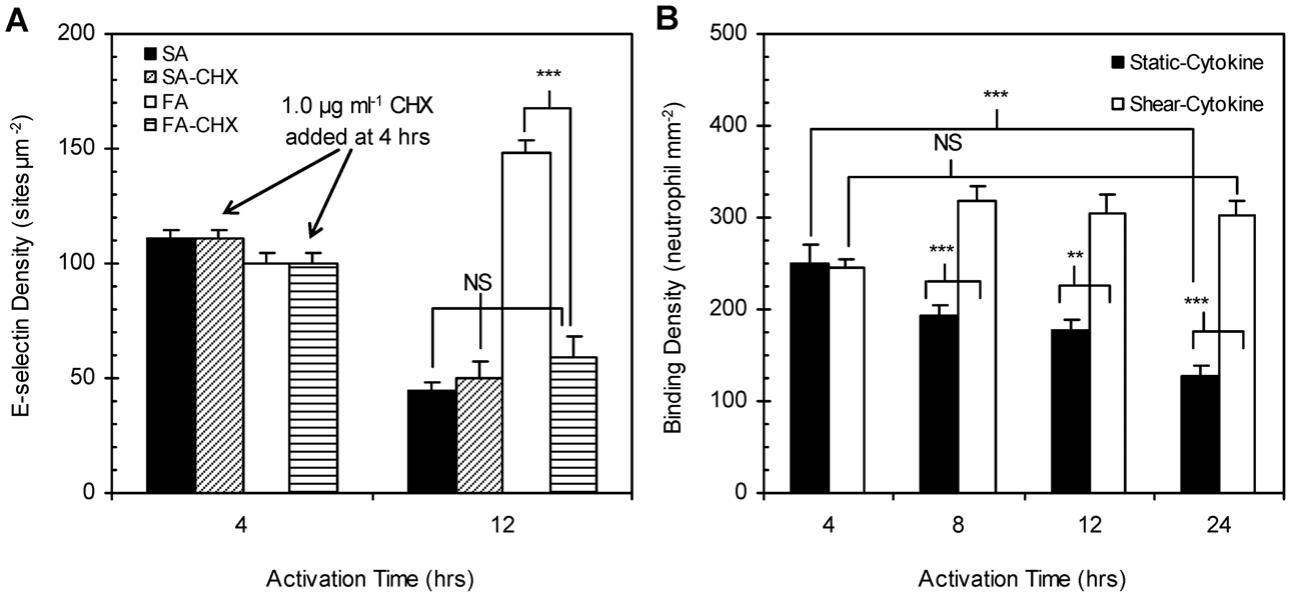
Cycloheximide (CHX) inhibition of E-selectin synthesis and neutrophil binding assays for static- and shear-cytokine activated monolayers. (A) E-selectin expression on cycloheximide-treated monolayers. CHX at a non-lethal concentration of 1 µg mL^−1^ was added to HUVEC monolayers at 4 hr activation time in both static (“SA-CHX,” diagonal stripes) and shear-cytokine (10 dyn cm^−2^, “FA-CHX,” horizontal stripes) conditions. Static (“SA,” solid) and shear-cytokine (“FA,” white) controls were not treated with CHX. (B) Neutrophil binding density over HUVEC monolayers activated with 0.1 ng ml^−1^ IL-1ß under static culture or 10 dyn cm^−2^ shear for 4, 8, 12, and 24 hr periods. “NS” indicates no significance, “***,” *p*<0.001, and “**,” *p*<0.05.

To determine whether upregulation (surface expression) of new E-selectin molecules rather than altered downregulation of previously expressed molecules contributed to the higher density observed with shear-cytokine activation at the 8–12 hr mark relative to static-cytokine, experiments were conducted with a non-lethal concentration of cycloheximide (CHX) (see Figure S3), an inhibitor of protein synthesis in eukaryotes, added to the shear-activation media at the 4 hr activation time point. Specifically, CHX was added to the media (at 1.0 µg mL^−1^) over EC monolayers that had been activating for 4 hr in static or shear conditions (thus CHX only affected protein expression beyond 4 hr). Monolayers treated with CHX remained under static- or shear-cytokine activation for an additional 8 hr period. E-selectin levels were quantified at the 12 hr activation mark. As shown in Figure 3A, the 12 hr E-selectin expression for monolayers exposed to shear-cytokine activation in the presence of CHX was significantly lower than values measured for monolayers exposed to shear-cytokine activation alone but was not significant from values measured for static-cytokine activation in the presence or absence of CHX. This indicates that the higher expression measured for EC exposed to shear-cytokine activation is due to additional (new) E-selectin being expressed on the apical surface and not delayed/altered downregulation.

The functionality of shear-cytokine-induced E-selectin molecules was evaluated relative to static-cytokine via neutrophil flow adhesion assays since E-selectin expression level on endothelial cells is known to correlate with the level of leukocyte adhesion during inflammation response [35], [36]. As shown in Figure 3B, HUVEC monolayers subjected to shear-cytokine activation at 10 dyn cm^−2^ supported significantly higher levels of neutrophil adhesion at the 8–24 hr activation time points when compared with monolayers activated under static conditions (1.6–2.4 folds higher). Monolayers exposed to static-cytokine activation showed maximum neutrophil binding after 4 hr activation and nearly half that value for the 24 hr activation. Neutrophil binding densities were similar between static- and shear-cytokine activation at the 4 hr time point in agreement with protein expression data.

### Effect of Shear Preconditioning on Shear-Cytokine Activation

**Figure 4:**
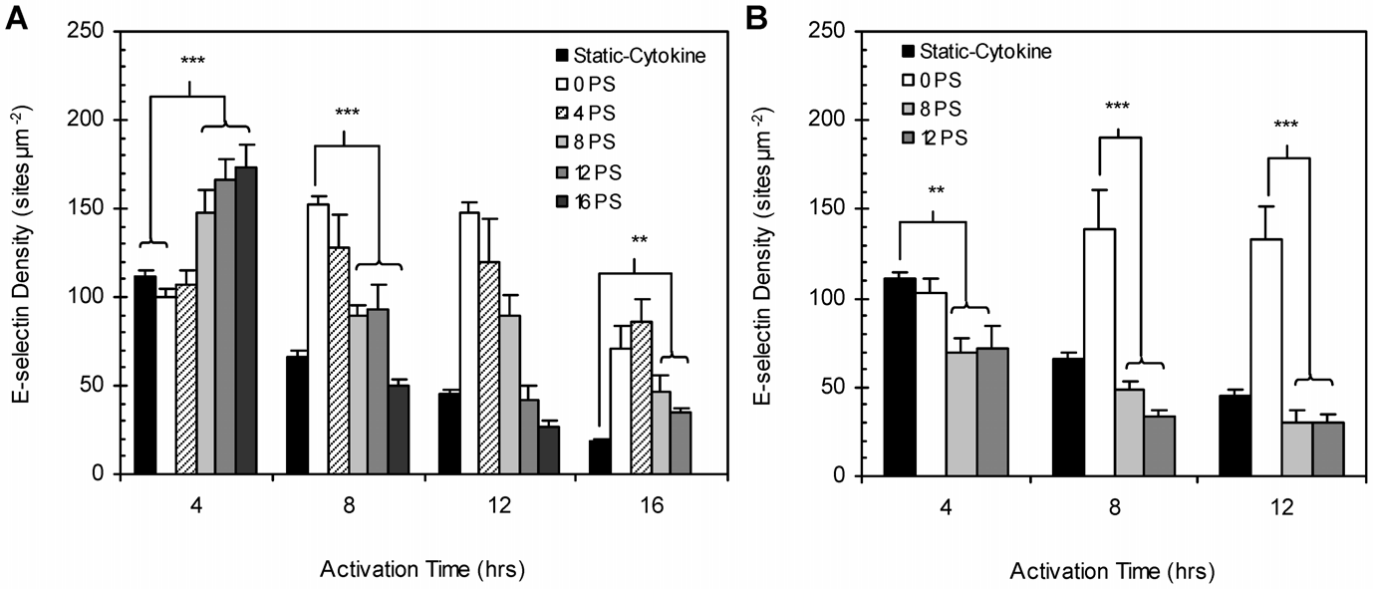
E-selectin expression (sites µm^−2^) on shear preconditioned monolayers. Site density on HUVECs preconditioned with (A) 10 dyn cm^−2^ or (B) 1 dyn cm^−2^ of laminar shear for 4, 8, 12, 16 or 20 hr (“4PS,” “8PS,” “12PS,” “16PS,” and “20PS,” respectively) prior to activation with 0.1 ng ml^−1^ IL-1ß under similar magnitude of shear. Filled bar = static activation controls and clear bar="0 PS” or monolayers were not preconditioned. “NS” indicates no significance, “***,” *p*<0.001, and “**,” *p*<0.05.

To more accurately represent physiological conditions, monolayers were preconditioned with low (1 dyn cm^−2^) or high laminar fluid shear (10 dyn cm^−2^) for up to 20 hr prior to shear-cytokine activation (at same shear magnitude). As shown in Figure 4A, ECs preconditioned for 4 hr with high shear prior to shear-cytokine activation displayed E-selectin expression levels comparable to naïve shear-cytokine activated monolayers (“0 PS”) with maximum expression occurring at 8–12 hr post activation. However, monolayers exposed to high shear preconditioning for 8, 12, and 16 hr prior to a 4 hr of shear-cytokine activation expressed E-selectin at levels 1.3–1.5 folds higher than naïve monolayers activated in static or shear-cytokine (controls) conditions for 4 hr. A further increase in preconditioning time to 20 hr, however, resulted in a significant drop in E-selectin expression in response to 4 hr of shear-cytokine activation relative to shear cytokine (naïve and preconditioned) and static activated monolayers at this shear-cytokine activation time point. Beyond the 4 hr shear-cytokine activation time point, all preconditioned monolayers exhibited E-selectin at levels lower than their corresponding shear-cytokine control. Monolayers subjected to 8 hr preconditioning, however, expressed higher levels of protein (2–2.6 folds higher) relative to static-cytokine control at all shear-cytokine activation times greater than 4 hr. E-selectin levels on monolayers preconditioned for 12 hr remained elevated against static-cytokine activation (1.4 fold higher) at 8 hr shear-cytokine activation whereas the level on 16 hr preconditioned monolayers did not. E-selectin expression on both 12 and 16 hr preconditioned monolayers dropped to levels not significant from the static-cytokine control at the 12 hr shear-cytokine activation mark, but both remained significantly different from static at the 16 hr activation mark.

Contrary to observations for high shear preconditioning, monolayers preconditioned for 8 or 12 hr with 1 dyn cm^−2^ of laminar shear (Figure 4B) showed a muted response to shear-cytokine activation at the same magnitude of shear, displaying significantly lower E-selectin expression when compared to the corresponding shear-cytokine activated controls at all activation times.

### Effect of Cytokine Redosing

**Figure 5:**
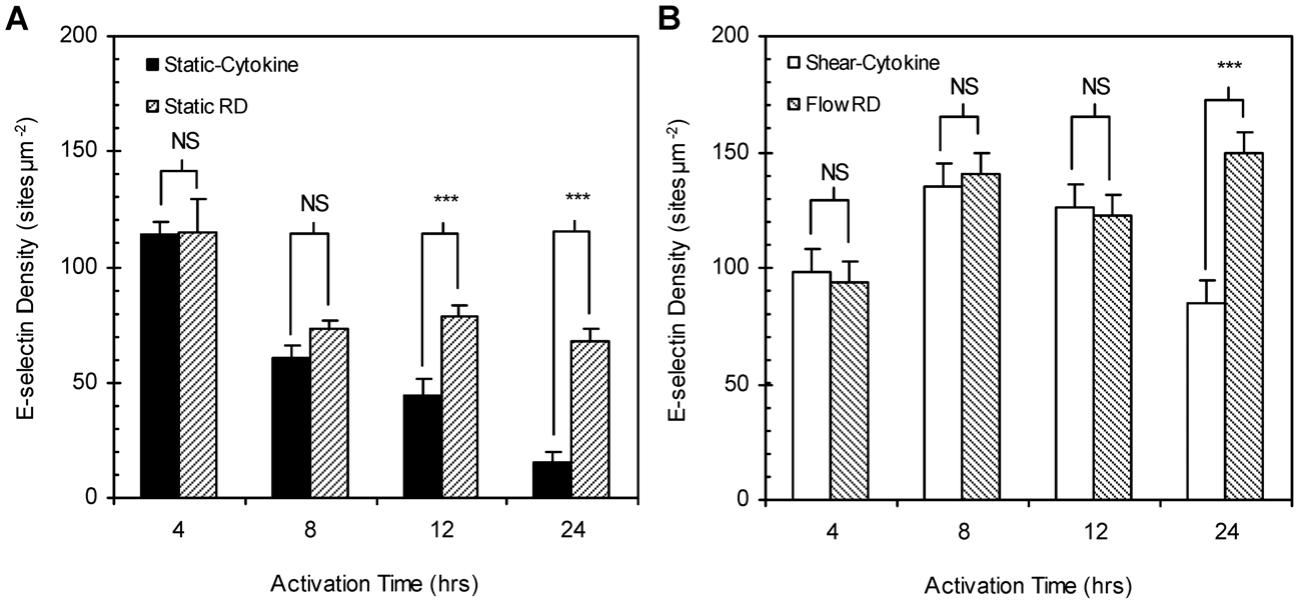
E-selectin expression (sites µm^−2^) on IL-1ß redosed monolayers. Redosing HUVEC monolayers in (A) static-cytokine or (B) shear-cytokine activation conditions. Control groups (filled and clear) were given only a single initial IL-1ß dose at the start of experiment. Redosed samples (upward and downward diagonals) were given repeated doses of IL-1ß every 4 hr until immunofluorescence assay at 8, 12, or 24 hr total activation time. Indicated significance values are comparing redosing and control data at the same timepoint. “NS” indicates no significance and “***,” *p*<0.001.

**Figure 6:**
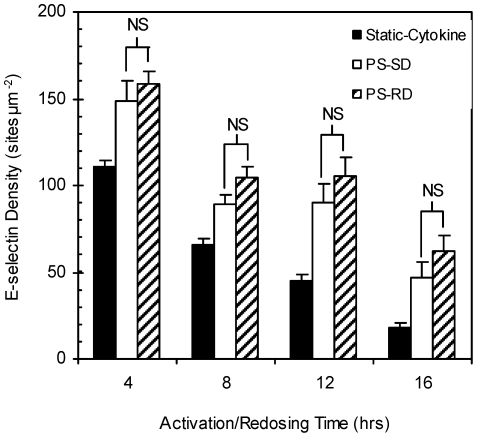
E-selectin expression (sites µm^−2^) on preconditioned and IL-1ß redosed monolayers. Site density of pre-conditioned HUVECs subjected to 4 hr intervals of IL-1ß redosing. “Static-Cytokine” (filled) monolayers were activated with initial IL-1ß dose at 0 hrs under static conditions; “PS-SD” (clear) monolayers were preconditioned under 10 dyn cm^−2^ of shear alone for 8 hrs prior to a single IL-1ß dose (under continued shear exposure); “PS-RD” (diagonal stripes) monolayers were preconditioned for 8 hrs with shear alone and then given repeated doses of IL-1ß (under continued shear exposure) every 4 hrs for up to 16 hrs total activation time. “NS” indicates no significance.

In order to simulate chronic inflammation (maintained exposure to a constant IL-1ß concentration), monolayers were subjected to multiple doses (redosing hereafter) of IL-1ß over time. Specifically, HUVEC monolayers were activated with an initial 0.1 ng ml^−1^ IL-1ß dose under static or fluid shear (10 dyn cm^−2^) conditions. Every four hours, spent culture media was removed from cells and replaced with fresh media containing the initial dose of IL-1ß. Monolayers redosed in static- or shear-cytokine activation conditions expressed similar E-selectin densities when compared to non-redosed controls at the 4 and 8 hr activation times as shown in Figure 5. Static-cytokine treated monolayers show a significant response to redosing at the 12 and 24 hr marks with higher E-selectin expression than non-redosed samples (1.8 and 5.2 fold higher, *p*<0.01) whereas shear-cytokine treated cells only showed a significant difference between redosed and control samples (1.7 fold higher, *p*<0.01) at the 24 hr mark. The effect of redosing on shear preconditioned ECs was also studied. For monolayers exposed to 8 hr shear preconditioning at 10 dyn cm^−2^ followed by shear-cytokine activation for up to 16 hr, E-selectin expression was not significantly different between monolayers redosed with IL-1ß every 4 hr and ones subjected to only an initial dose of IL-1ß at 0 hr as shown in Figure 6.

## Discussion

The endothelium plays critical roles in the regulation of natural processes as well as pathological events such as chronic inflammation and cancer metastasis. Thus, understanding the differential response of endothelial cells (ECs) to various agonists, including cytokines and mechanical forces imparted by blood flow, has long been a focus of biomedical research. In inflammation response, the endothelium modulates the margination of blood leukocytes via differential expression of leukocyte adhesion molecules with the selectins (E and P) being important for their initial adhesive interactions. The ability to identify and quantify the expression patterns for these markers is invaluable for the design of sophisticated vascular-targeted diagnostics or therapeutic systems useful in the many diseases that chronic inflammation is known to have a role. *In vitro* assays with human ECs are critical to the aforementioned prospect since tools that allow for the imaging of protein expression *in vivo* are currently lacking. One major drawback of previously described *in vitro* models, especially in attempting to represent a system as dynamic as the vascular tissue, is in not adequately simulating the physiological conditions under which inflammation occurs. Specifically, only a few works in the literature have explored any cytokine activation of cultured ECs in the presence of physiologically relevant shear and even fewer with IL-1ß and shear. However, it is well-known that ECs are highly responsive to mechanical forces by way of morphological alignment (*e.g.* transitioning from cobblestone to elongated shapes after a period of laminar shear [43], [44], [45]) and surface expression of some leukocyte adhesion proteins [46]. In the current study, we investigated EC response to long-term inflammatory cues under physiologically relevant shear conditions where monolayers of human umbilical vein endothelial cells (HUVECs) were exposed to simultaneous stimulation of laminar fluid shear and IL-1ß.

We show for the first time that the co-stimulation of naïve ECs (not previously exposed to shear *in vitro*) with flow-induced shear and IL-1β induces the expression of significantly higher levels of functional E-selectin molecules up to 24 hr when compared with monolayers stimulated under static condition only (Figure 1). This is interesting since E-selectin expression by ECs is typically not inducible by laminar shear alone [35], [47], [48] as was shown in Figure 1 though one report showed a 4 fold increase in E-selectin mRNA in human aortic ECs in response to 4.5 dyn cm^−2^ (but not at >10 dyn cm^−2^) of laminar shear [49]. Similarly, peak E-selectin expression is well-documented to occur between 4–6 hr in static-cytokine stimulated cells compared to the 8–12 hr peak observed with naïve cells under shear-cytokine activation (Figure 2A). As shown in Figure 5B, maximal E-selectin expression was extended to 24 hr when cytokine redosing is incorporated. The observed lack of EC response to IL-1β redosing at earlier time points (4 and 8 hrs for static and up to 12 hr for shear-cytokine) is in line with previous report by Pober *et al.* [15] where EC monolayers in static were shown to express E-selectin levels at 30 hr that were comparable to the 4 hr peak expression for a single dose only if the redosing occurred at 24 hr for monolayers initially dosed for six hours and rested (removal of cytokine) for eighteen hours. The authors showed that redosing at the 24 hr mark of ECs that had been under continuous presence of an initial IL-1β dose yielded no response at the 30 hr mark. Thus, it is possible that the multiple 4 hr-interval redosing as presented in this work conditioned ECs to better respond with higher E-selectin expression at the 12 and 24 hr mark for static- and shear-cytokine activation, respectively.

From the protein translation inhibition data presented in Figure 3A, we conclude that the higher density of E-selectin on shear-cytokine activated naïve ECs is likely associated with increased surface expression of E-selectin rather than due to a decrease in the E-selectin internalization rate. A previous report by Kluger *et al.* that found E-selectin internalization rate on HUVEC to be constant regardless of expression level further supports this conclusion [50], and Kraiss *et al.* showed that shear stress modulates TNF-α-induced expression of E-selectin at the protein translation level [51]. The shear-cytokine newly-induced E-selectin were found to be functional for leukocyte recruitment since neutrophil adhesion density on shear-cytokine activated monolayers relative to static-cytokine activated monolayers was found to directly correlate to protein expression patterns as shown in Figure 3B [36]. Interestingly, E-selectin expression under shear-cytokine conditions appears to be insensitive to the magnitude of shear imposed during shear-cytokine activation at most of the time points studied (Figure 2). A shear threshold of 2 dyn cm^−2^ was required at the 24 hr activation time to induce the same 3 folds increase in E-selectin expression by naïve ECs at intermediate activation times for shear-cytokine activation relative to static-cytokine activation.

While at first glance, the E-selectin expression profile that emerged for monolayers preconditioned at a high laminar shear prior to shear-cytokine activation (Figure 4A) appears contradictory to the profile observed for naïve cells in shear-cytokine activation, a closer look suggests one similarity between the two. Specifically, in both naïve and shear-conditioned ECs, the optimum protein expression in response to shear-cytokine activation occurs after cells had been exposed to at least eight hours of flow-induced shear. This time frame coincides with that for which naïve EC monolayers were previously observed to realign and elongate in the direction of flow in response to shear (see Figure S2) [52], [53]. This observation further highlights the active role that cell cytoskeleton may have in regulating E-selectin protein expression as previously suggested by others [54], [55].

Consistent with previous literature, the presented data shows that laminar shear stress is protective against chronic inflammation (>24 hr) in ECs that have been subjected to a prolonged period of high shear prior to cytokine stimulation as would exist in physiological occurrences of normal inflammation response [56], [57]. Most interesting, however, is the observation in Figure 4A that naïve cells exposed to high shear prior to cytokine exposure under shear conditions exhibited markedly higher E-selectin expression in the first four hours of shear-cytokine activation relative to naïve cells activated under static- or shear-cytokine conditions. This phenomenon is not simply attributed to cumulative shear exposure time as, for example, monolayers preconditioned for 12 hr followed by 4 hr of shear-cytokine activation exhibited ∼2.5 fold higher E-selectin expression compared to naïve cells exposed to 16 hr of shear-cytokine stimulation. A minimum shear-preconditioning magnitude and time threshold seems to be a requisite for this observation: higher E-selectin expression relative to the 4 hr static-cytokine activation was absent in monolayers preconditioned with low laminar shear (1 dyn cm^−2^; Figure 4B) or for monolayers preconditioned for times outside the 8–16 hr range. It is also interesting to note that redosing of preconditioned ECs with fresh IL-1β during shear-cytokine activation did not induce higher E-selectin expression even at prolonged shear-cytokine activation times (Figure 6) whereas a positive effect was observed in redosing of naïve cells (Figure 5) particularly at the 24 hr mark. This highlights the robust protection against chronic inflammation conferred by prior exposure to high laminar shear, *i.e.* the individual sustained synthesis or continual presence of an elevated concentration of cytokine alone is not enough to induce chronic expression of E-selectin *in vivo* in healthy vessels with high laminar shear. One focus of our future work would be to explore any potential synergy between multiple cytokines (*e.g.* IL-1β and TNF-α) and flow-induced shear in the temporal EC expression of E-selectin and other leukocyte adhesion molecules.

It is worth noting, however, that the observed shear-regulated differential E-selectin expression may be cytokine-specific since a previous report of TNF-α activation of shear preconditioned ECs resulted in an expression level that was not significantly different from that observed in unactivated naïve ECs in static, contrary to the data reported here [24]. The same authors later showed minimal neutrophil adhesion on HUVEC preconditioned with high shear for 24 hr followed by a 3 hr TNF-α activation [21]. This discrepancy between EC response to IL-1β and TNF-α activation in shear may be due to their distinct cell surface receptors that effect slightly different EC signaling pathways [15]. However, Cicha *et al.* and Chiu *et al.* each independently showed that HUVECs subjected to 18 and 24 hr of high shear preconditioning followed by 2 and 4 hr of shear-cytokine (TNF-α) activation, respectively, expressed E-selectin at levels significantly higher than the unactivated controls with the latter study showing a 4 fold lower E-selectin expression relative to static-cytokine activated naïve ECs [22], [23]. The conflicting reports on the effect of shear preconditioning on EC response to TNF-α stimulation is likely only resolved via a similar comprehensive analysis of EC response to the combined shear-TNF-α stimulation as is presented here for shear-IL-1β. Our future work will address this.

In summary, we report for the first time that human EC expression of E-selectin in response to IL-1β stimulation is dependent on the shear history of these cells. Naïve cells simultaneously exposed to IL-1β and high laminar shear display strong pro-inflammatory phenotypes for an extended period whereas cells first preconditioned with laminar shear exhibit either elevated or muted inflammatory response dependent on the time frame of the following shear-cytokine stimulation. While the shear-cytokine stimulation of naïve or short-term preconditioned cells (<24 hr) and their ensuing E-selectin expression pattern may not be relevant in normal inflammation response, the presented data likely provides insight into the pathological inflammatory response in ischemia since IL-1β is known to be strongly involved in the pathogenesis of ischemic brain damage and other acute and chronic neurodegenerative disorders[58], [59]. For example, the elongated endothelial morphology conferred by flow is known to be reversible upon cessation of flow [41], [42]; thus the endothelium downstream of a blood vessel obstruction or constriction (as seen in atherosclerosis or thromboembolism) may acquire a naïve-like phenotype dependent on the extent of ischemia. These ECs may then display a hyper-inflammatory phenotype and the presented E-selectin expression pattern (Figure 1) upon reperfusion wherein cytokine present at the obstruction is delivered simultaneously with flow induced-shear to downstream endothelial cells. Thus, it is possible that an enhanced inflammatory EC phenotype (and hence, elevated E-selectin expression) during flow reperfusion in the presence of IL-1β in part contributes to the high level of leukocyte recruitment that has been suggested as a cause of the phenomenon of reperfusion injury [60], [61]. This hypothesis may be further substantiated by evidence of increased E-selectin expression in microvessels in ischemic lesions after 4–24 hr of reperfusion [62], [63]. In one study, peak E-selectin expression was measured after 10 hr of reperfusion consistent with the time frame of the highest E-selectin expression by naïve and preconditioned ECs as reported here (Figures 2 and 4) [63]. Evidence also exists that the endothelial dysfunction associated in the reperfusion phase is decoupled from any injury associated with the preceding ischemia [64], [65]. However, the use of HUVECs in this study may limit the direct correlation of E-selectin expression to clinically relevant ischemia/reperfusion injury. Our future work will explore protein expression patterns in human ECs from other vascular beds more prone to developing pathologies in response to various combinations of steady and disturbed flow patterns and cytokine stimulation.

## Supporting Information

Figure S1 **E-selectin expression on endothelial monolayer at 4 hrs static activation.** HUVECs were exposed to 0.1 ng ml^−1^ IL-1ß under static conditions for 4 hrs. A fluorescent (A) and brightfield (B) image of E-selectin labeled with FITC-conjugated anti-human E-selectin antibody was captured at 10× magnification and overlaid to create a composite image (C).
(TIFF)

Figure S2 **Images of endothelial morphology over long-term shear-cytokine activation.** Brightfield images at 20× magnification of HUVECs exposed to 0.1 ng ml^−1^ IL-1ß under 10 dyn cm^−2^ shear for (A) 0 hr, (B) 4 hr, (C) 8 hr, and (D) 24 hr.
(TIFF)

Figure S3 **Endothelial monolayer integrity after treatment with cylcoheximide and IL-1β.** Monolayers were simultaneously treated with 1.0 µg ml^−1^ of cycloheximide and 0.1 ng ml^−1^ IL-1ß in growth media. Brightfield images (20×) were captured at (A) 4 hrs and (B) 8 hrs.
(TIFF)

## References

[1] Coussens LM, Werb Z (2002). Inflammation and cancer.. Nature.

[2] Davies PF, Shi C, Depaola N, Helmke BP, Polacek DC (2001). Hemodynamics and the focal origin of atherosclerosis: a spatial approach to endothelial structure, gene expression, and function.. Ann N Y Acad Sci.

[3] Erdman SE, Poutahidis T (2010). Cancer inflammation and regulatory T cells.. Int J Cancer.

[4] Jang IK, Lassila R, Fuster V (1993). Atherogenesis and inflammation.. Eur Heart J.

[5] Libby P (2007). Inflammatory mechanisms: the molecular basis of inflammation and disease.. Nutr Rev.

[6] Tiong AY, Brieger D (2005). Inflammation and coronary artery disease.. Am Heart J.

[7] Aird WC (2008). Endothelium in health and disease.. Pharmacol Rep.

[8] Pries AR, Kuebler WM (2006). Normal endothelium.. Handb Exp Pharmacol.

[9] Shimokawa H (1999). Primary endothelial dysfunction: atherosclerosis.. J Mol Cell Cardiol.

[10] Thorin E, Shreeve SM (1998). Heterogeneity of vascular endothelial cells in normal and disease states.. Pharmacol Ther.

[11] Amberger A, Maczek C, Jurgens G, Michaelis D, Schett G (1997). Co-expression of ICAM-1, VCAM-1, ELAM-1 and Hsp60 in human arterial and venous endothelial cells in response to cytokines and oxidized low-density lipoproteins.. Cell Stress Chaperones.

[12] Hanada T, Yoshimura A (2002). Regulation of cytokine signaling and inflammation.. Cytokine Growth Factor Rev.

[13] Henninger DD, Panes J, Eppihimer M, Russell J, Gerritsen M (1997). Cytokine-induced VCAM-1 and ICAM-1 expression in different organs of the mouse.. J Immunol.

[14] Kluger MS, Johnson DR, Pober JS (1997). Mechanism of sustained E-selectin expression in cultured human dermal microvascular endothelial cells.. J Immunol.

[15] Pober JS, Bevilacqua MP, Mendrick DL, Lapierre LA, Fiers W (1986). Two distinct monokines, interleukin 1 and tumor necrosis factor, each independently induce biosynthesis and transient expression of the same antigen on the surface of cultured human vascular endothelial cells.. J Immunol.

[16] DePaola N, Gimbrone MA, Davies PF, Dewey CF (1992). Vascular endothelium responds to fluid shear stress gradients.. Arterioscler Thromb.

[17] Gimbrone MA, Topper JN, Nagel T, Anderson KR, Garcia-Cardena G (2000). Endothelial dysfunction, hemodynamic forces, and atherogenesis.. Ann N Y Acad Sci.

[18] Imberti B, Seliktar D, Nerem RM, Remuzzi A (2002). The response of endothelial cells to fluid shear stress using a co-culture model of the arterial wall.. Endothelium.

[19] Yee A, Sakurai Y, Eskin SG, McIntire LV (2006). A validated system for simulating common carotid arterial flow in vitro: alteration of endothelial cell response.. Ann Biomed Eng.

[20] Conway DE, Williams MR, Eskin SG, McIntire LV (2010). Endothelial cell responses to atheroprone flow are driven by two separate flow components: low time-average shear stress and fluid flow reversal.. Am J Physiol Heart Circ Physiol.

[21] Matharu NM, McGettrick HM, Salmon M, Kissane S, Vohra RK (2008). Inflammatory responses of endothelial cells experiencing reduction in flow after conditioning by shear stress.. J Cell Physiol.

[22] Chiu JJ, Lee PL, Chen CN, Lee CI, Chang SF (2004). Shear stress increases ICAM-1 and decreases VCAM-1 and E-selectin expressions induced by tumor necrosis factor-[alpha] in endothelial cells.. Arterioscler Thromb Vasc Biol.

[23] Cicha I, Beronov K, Ramirez EL, Osterode K, Goppelt-Struebe M (2009). Shear stress preconditioning modulates endothelial susceptibility to circulating TNF-alpha and monocytic cell recruitment in a simplified model of arterial bifurcations.. Atherosclerosis.

[24] Sheikh S, Rainger GE, Gale Z, Rahman M, Nash GB (2003). Exposure to fluid shear stress modulates the ability of endothelial cells to recruit neutrophils in response to tumor necrosis factor-alpha: a basis for local variations in vascular sensitivity to inflammation.. Blood.

[25] Dinarello CA (2011). Blocking interleukin-1beta in acute and chronic autoinflammatory diseases.. Journal of internal medicine.

[26] Griffin WS, Mrak RE (2002). Interleukin-1 in the genesis and progression of and risk for development of neuronal degeneration in Alzheimer's disease.. Journal of leukocyte biology.

[27] Dinarello CA (2009). Immunological and inflammatory functions of the interleukin-1 family.. Annual review of immunology.

[28] Maedler K, Dharmadhikari G, Schumann DM, Storling J (2011). Interleukin-targeted therapy for metabolic syndrome and type 2 diabetes.. Handbook of experimental pharmacology.

[29] Nagel T, Resnick N, Atkinson WJ, Dewey CF, Gimbrone MA (1994). Shear stress selectively upregulates intercellular adhesion molecule-1 expression in cultured human vascular endothelial cells.. J Clin Invest.

[30] Ley K, Laudanna C, Cybulsky MI, Nourshargh S (2007). Getting to the site of inflammation: the leukocyte adhesion cascade updated.. Nat Rev Immunol.

[31] Parekh RB, Edge CJ (1994). Selectins–glycoprotein targets for therapeutic intervention in inflammation.. Trends in biotechnology.

[32] Galkina E, Ley K (2007). Leukocyte influx in atherosclerosis.. Current drug targets.

[33] Ferrario CM, Strawn WB (2006). Role of the renin-angiotensin-aldosterone system and proinflammatory mediators in cardiovascular disease.. The American journal of cardiology.

[34] Roldan V, Marin F, Lip GY, Blann AD (2003). Soluble E-selectin in cardiovascular disease and its risk factors. A review of the literature.. Thrombosis and haemostasis.

[35] Morigi M, Zoja C, Figliuzzi M, Foppolo M, Micheletti G (1995). Fluid shear stress modulates surface expression of adhesion molecules by endothelial cells.. Blood.

[36] Lei YP, Chen HW, Sheen LY, Lii CK (2008). Diallyl disulfide and diallyl trisulfide suppress oxidized LDL-induced vascular cell adhesion molecule and E-selectin expression through protein kinase A- and B-dependent signaling pathways.. The Journal of nutrition.

[37] Burns AR, Bowden RA, MacDonell SD, Walker DC, Odebunmi TO (2000). Analysis of tight junctions during neutrophil transendothelial migration.. Journal of cell science.

[38] Charoenphol P, Huang RB, Eniola-Adefeso O (2010). Potential role of size and hemodynamics in the efficacy of vascular-targeted spherical drug carriers.. Biomaterials.

[39] Eniola-Adefeso O, Huang RB, Smith CW (2009). Kinetics of LFA-1 mediated adhesion of human neutrophils to ICAM-1–role of E-selectin signaling post-activation.. Ann Biomed Eng.

[40] Charoenphol P, Mocherla S, Bouis D, Namdee K, Pinsky DJ (2011). Targeting therapeutics to the vascular wall in atherosclerosis-Carrier size matters.. Atherosclerosis.

[41] Malek AM, Izumo S (1996). Mechanism of endothelial cell shape change and cytoskeletal remodeling in response to fluid shear stress.. J Cell Sci.

[42] Remuzzi A, Dewey CF, Davies PF, Gimbrone MA (1984). Orientation of endothelial cells in shear fields in vitro.. Biorheology.

[43] Dewey CF, Bussolari SR, Gimbrone MA, Davies PF (1981). The dynamic response of vascular endothelial cells to fluid shear stress.. J Biomech Eng.

[44] Ives CL, Eskin SG, McIntire LV (1986). Mechanical effects on endothelial cell morphology: in vitro assessment.. In Vitro Cell Dev Biol.

[45] Levesque MJ, Nerem RM (1985). The elongation and orientation of cultured endothelial cells in response to shear stress.. J Biomech Eng.

[46] Li M, Scott DE, Shandas R, Stenmark KR, Tan W (2009). High pulsatility flow induces adhesion molecule and cytokine mRNA expression in distal pulmonary artery endothelial cells.. Ann Biomed Eng.

[47] Ji JY, Jing H, Diamond SL (2008). Hemodynamic regulation of inflammation at the endothelial-neutrophil interface.. Ann Biomed Eng.

[48] Nagel T, Resnick N, Atkinson WJ, Dewey CF, Gimbrone MA (1994). Shear stress selectively upregulates intercellular adhesion molecule-1 expression in cultured human vascular endothelial cells.. The Journal of clinical investigation.

[49] Rouleau L, Rossi J, Leask RL (2010). The response of human aortic endothelial cells in a stenotic hemodynamic environment: effect of duration, magnitude, and spatial gradients in wall shear stress.. Journal of biomechanical engineering.

[50] Kluger MS, Shiao SL, Bothwell AL, Pober JS (2002). Cutting Edge: Internalization of transduced E-selectin by cultured human endothelial cells: comparison of dermal microvascular and umbilical vein cells and identification of a phosphoserine-type di-leucine motif.. J Immunol.

[51] Kraiss LW, Alto NM, Dixon DA, McIntyre TM, Weyrich AS (2003). Fluid flow regulates E-selectin protein levels in human endothelial cells by inhibiting translation.. Journal of vascular surgery : official publication, the Society for Vascular Surgery [and] International Society for Cardiovascular Surgery, North American Chapter.

[52] Langille BL, Graham JJ, Kim D, Gotlieb AI (1991). Dynamics of shear-induced redistribution of F-actin in endothelial cells in vivo.. Arterioscler Thromb.

[53] Noria S, Cowan DB, Gotlieb AI, Langille BL (1999). Transient and steady-state effects of shear stress on endothelial cell adherens junctions.. Circ Res.

[54] Kuijpers TW, Raleigh M, Kavanagh T, Janssen H, Calafat J (1994). Cytokine-activated endothelial cells internalize E-selectin into a lysosomal compartment of vesiculotubular shape. A tubulin-driven process.. J Immunol.

[55] Matteoni R, Kreis TE (1987). Translocation and clustering of endosomes and lysosomes depends on microtubules.. J Cell Biol.

[56] Partridge J, Carlsen H, Enesa K, Chaudhury H, Zakkar M (2007). Laminar shear stress acts as a switch to regulate divergent functions of NF-kappaB in endothelial cells.. FASEB J.

[57] Yamawaki H, Lehoux S, Berk BC (2003). Chronic physiological shear stress inhibits tumor necrosis factor-induced proinflammatory responses in rabbit aorta perfused ex vivo.. Circulation.

[58] Allan SM, Tyrrell PJ, Rothwell NJ (2005). Interleukin-1 and neuronal injury.. Nat Rev Immunol.

[59] Boutin H, LeFeuvre RA, Horai R, Asano M, Iwakura Y (2001). Role of IL-1alpha and IL-1beta in ischemic brain damage.. J Neurosci.

[60] Carden DL, Smith JK, Korthuis RJ (1990). Neutrophil-mediated microvascular dysfunction in postischemic canine skeletal muscle. Role of granulocyte adherence.. Circ Res.

[61] Rodrigues SF, Granger DN (2010). Role of blood cells in ischaemia-reperfusion induced endothelial barrier failure.. Cardiovasc Res.

[62] Haring HP, Berg EL, Tsurushita N, Tagaya M, del Zoppo GJ (1996). E-selectin appears in nonischemic tissue during experimental focal cerebral ischemia.. Stroke.

[63] Zhang RL, Chopp M, Zhang ZG, Phillips ML, Rosenbloom CL (1996). E-selectin in focal cerebral ischemia and reperfusion in the rat.. J Cereb Blood Flow Metab.

[64] Inauen W, Payne DK, Kvietys PR, Granger DN (1990). Hypoxia/reoxygenation increases the permeability of endothelial cell monolayers: role of oxygen radicals.. Free Radic Biol Med.

[65] Parks DA, Granger DN (1986). Contributions of ischemia and reperfusion to mucosal lesion formation.. Am J Physiol.

