# Supplementary figures and images for: Shear Stress Modulation of IL-1β-Induced E-Selectin Expression in Human Endothelial Cells
Source: PLoS One. 2012 Feb 24;7(2):e31874. doi: 10.1371/journal.pone.0031874 (PMC3286450; doi:10.1371/journal.pone.0031874)

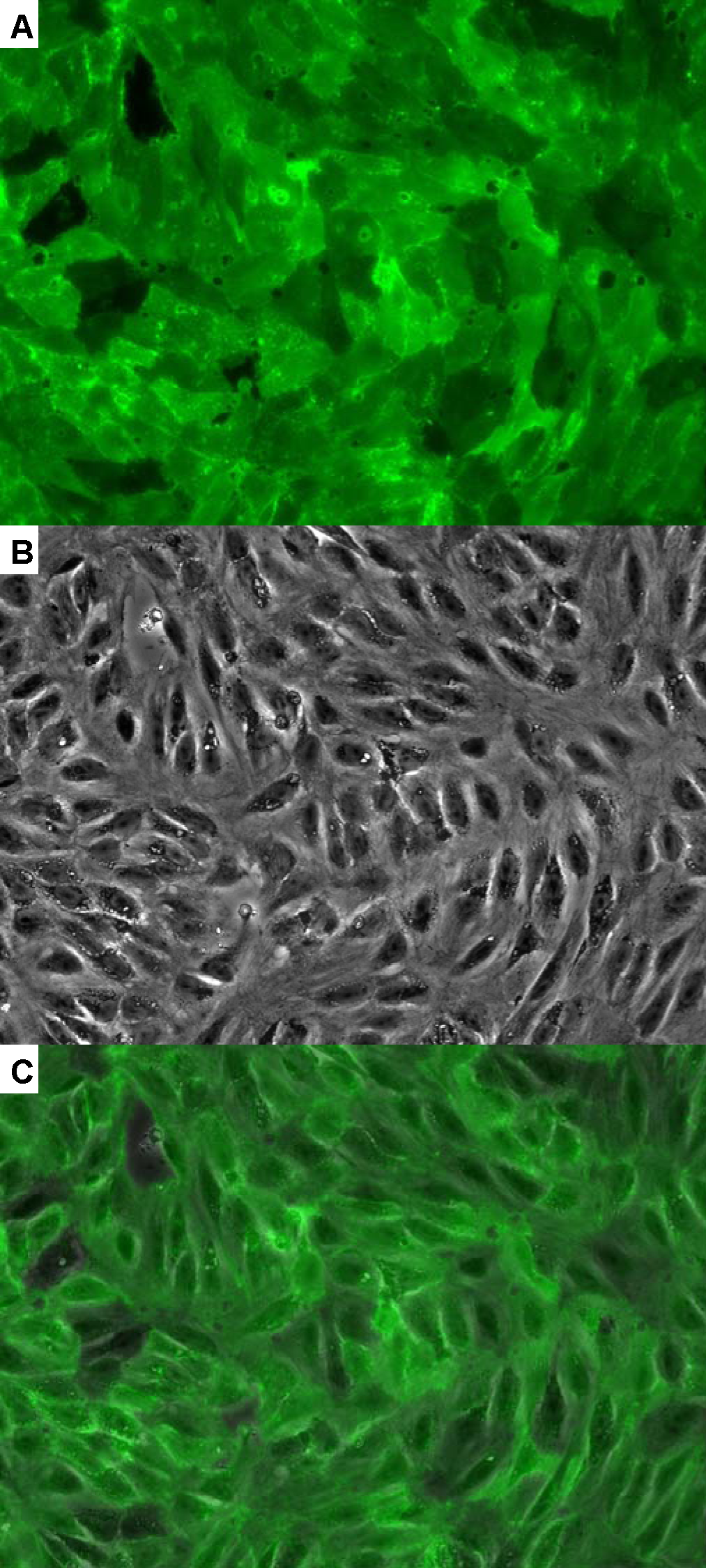

Supplement: Figure S1 — E-selectin expression on endothelial monolayer at 4 hrs static activation. HUVECs were exposed to 0.1 ng ml−1 IL-1ß under static conditions for 4 hrs. A fluorescent (A) and brightfield (B) image of E-selectin labeled with FITC-conjugated anti-human E-selectin antibody was captured at 10× magnification and overlaid to create a composite image (C). (TIFF) [file pone.0031874.s001.tif]

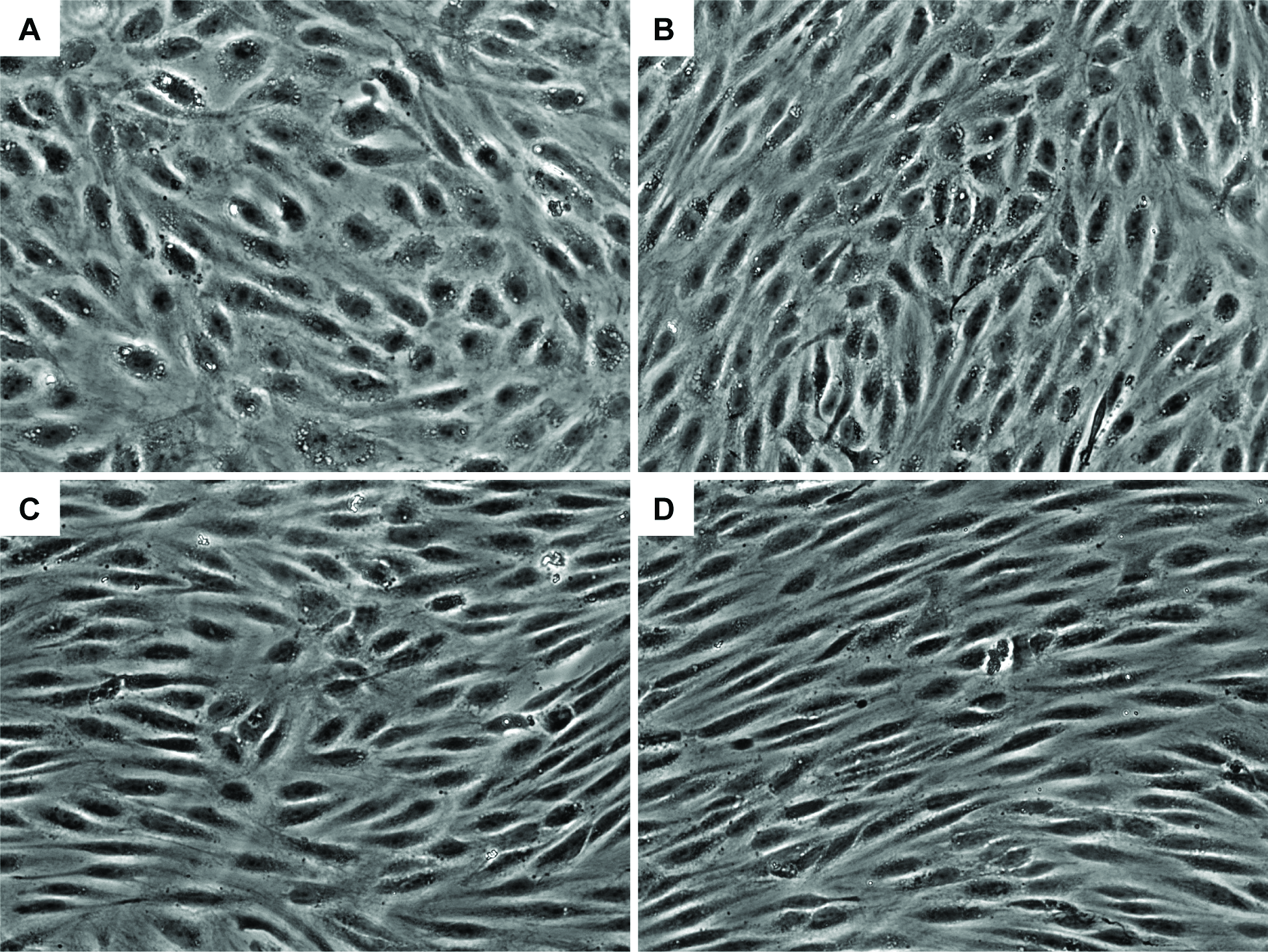

Supplement: Figure S2 — Images of endothelial morphology over long-term shear-cytokine activation. Brightfield images at 20× magnification of HUVECs exposed to 0.1 ng ml−1 IL-1ß under 10 dyn cm−2 shear for (A) 0 hr, (B) 4 hr, (C) 8 hr, and (D) 24 hr. (TIFF) [file pone.0031874.s002.tif]

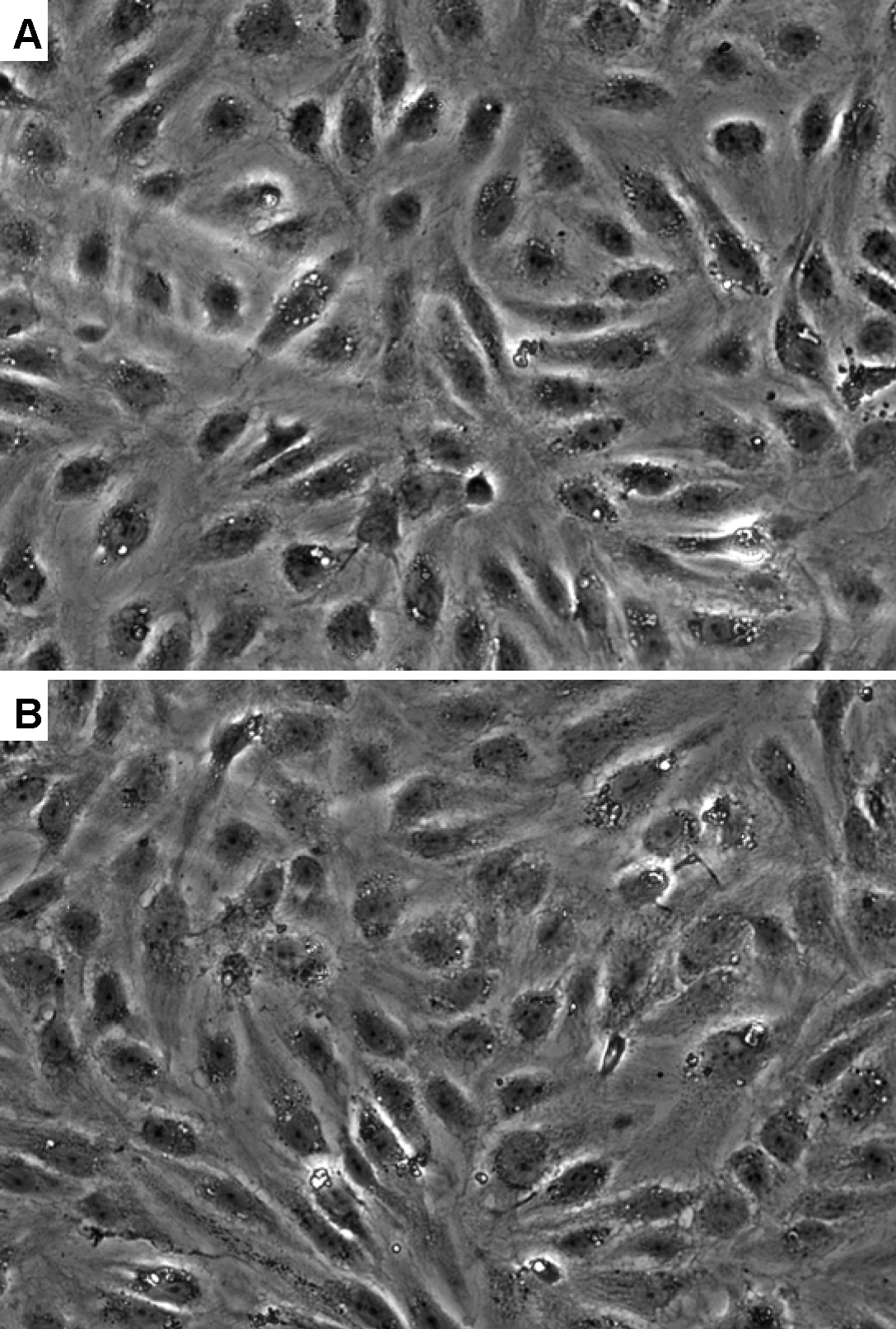

Supplement: Figure S3 — Endothelial monolayer integrity after treatment with cylcoheximide and IL-1β. Monolayers were simultaneously treated with 1.0 µg ml−1 of cycloheximide and 0.1 ng ml−1 IL-1ß in growth media. Brightfield images (20×) were captured at (A) 4 hrs and (B) 8 hrs. (TIFF) [file pone.0031874.s003.tif]
